# Supplementary material for: High-resolution analysis of bound Ca2+ in neurons and synapses
Source: Life Sci Alliance. 2023 Oct 13;7(1):e202302030. doi: 10.26508/lsa.202302030 (PMC10575792; doi:10.26508/lsa.202302030)
Supplement: Supplementary file 4 [file LSA-2023-02030_TableS1.pdf]

**Table S1.**

All nanobodies and primary and secondary antibodies used in this study.

| Name | Type | Company | Catalog No. | Ratio |
|---|---|---|---|---|
| Synaptotagmin (Oyster488) | Nanobody | Synaptic Systems | 105 311 C2 | 1:500 |
| FluoTag-x2 Anti-mouse (Atto542) | Nanobody | NanoTag | N1202-At54 | 1:500 |
| Synaptophysin | Antibody | Synaptic Systems | 101 004 | 1:1000 |
| VGLUT1 | Antibody | Synaptic Systems | 135 011 | 1:500 |
| FluoTag-x2 Anti-PSD95 (custom-conjugated to Cy3) | Nanobody | NanoTag | N3702 | 1:1000 |
| VGAT | Antibody | Synaptic Systems | 131 002 | 1:200 |
| Alexa488 (anti-mouse) | Antibody | Dianova | 715-545-151 | 1:100 |
| Star635P (anti-guinea pig) | Antibody | Abberior | ST635P-1006-500UG | 1:100 |
| Star635P (anti-rabbit) | Antibody | Abberior | ST635P-1002-500UG | 1:100 |
